# Online revision process in clause-boundary garden-path sentences

**DOI:** 10.3758/s13421-023-01444-0

**Published:** 2023-07-19

**Authors:** Hiroki Fujita

**Affiliations:** Department of Linguistics, University of Potsdam, Karl-Liebknecht-Str. 24–25, 14476 Potsdam, Germany

**Keywords:** Garden path, Revision, Structural ambiguity, Triage, Reflexive resolution, Relative clause, Sentence parsing, Language comprehension

## Abstract

A long-standing question in sentence processing research concerns the online parsing process in clause-boundary garden-path sentences, such as *After Mary dressed John bathed*. In this sentence, “John” must be parsed as the matrix subject DP but can be locally analysed as the object of the embedded verb. There is considerable evidence that the parser misanalyses these garden-path sentences. However, the controversy lies in whether the parser revises them during the online parsing process. The present study investigated this revision process through two self-paced reading experiments utilising grammatical constraints on reflexives and subject or object relative clauses embedded within the locally ambiguous DP. The results provided evidence of revision when a subject relative clause was embedded but not when an object relative clause was embedded. These findings suggest that the parser assigns grammatical structures that correspond to input strings during the revision of clause-boundary ambiguities but that object relative clauses affect the online revision process.

## Introduction

Online sentence comprehension may involve the incremental assignment of hierarchical syntactic structures to sentences (e.g., Chomsky, 1957; Crocker, 1996; Fujita, 2021b, 2023; Kimball, 1973; Matthews, 1961; Phillips, 1996; Sturt, 1997; Weinberg, 1999). During this parsing process, a string of words often corresponds to multiple grammatical structures simultaneously. Studies have indicated that the parser favours specific structures over others during sentence processing. This parsing preference has been demonstrated through the observation of processing difficulty (e.g., increased processing times), called *garden-path effects* (e.g., L. Frazier & Rayner, 1982), when subsequent input disambiguates ambiguities (*local ambiguities*; Abney & Johnson, 1991). Consider, for example, the following sentence.

- (1) After Mary dressed John bathed himself.
- (2a) [_CP_ [_PP_ After Mary dressed [_DP_ John]] [_TP_…]]^1^
- (2b) [_CP_ [_PP_ After Mary dressed] [_TP_ [_DP_ John]…]]

**Fig. 1:**
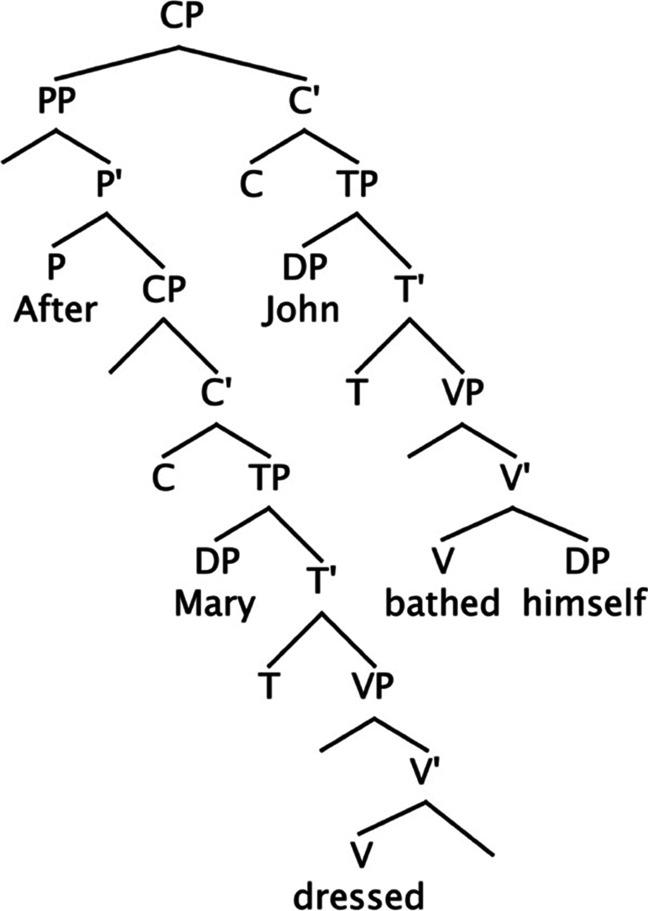
A hierarchical syntactic structure corresponding to the sentence in (1) in the text

**Fig. 2:**
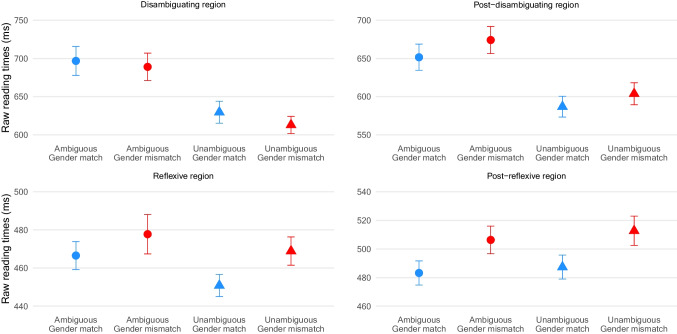
Mean reading times at the (post-)disambiguating and (post-)reflexive regions in Experiment 1. Error bars are standard errors

**Fig. 3:**
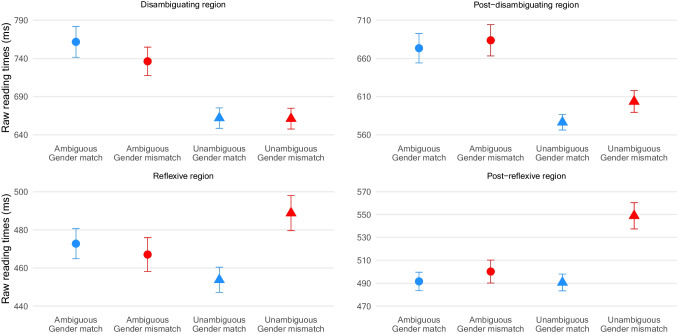
Mean reading times at the (post-)disambiguating and (post-)reflexive regions in Experiment 2. Error bars are standard errors

In (1), “John” must be parsed as a DP in the matrix subject position (*the matrix subject*), as represented in (2b). However, it can be locally analysed as the object of the verb in the embedded clause (*the embedded object*), as depicted in (2a), until the matrix verb “bathed” appears.^2^ There is considerable evidence that this *clause-boundary ambiguity* causes garden-path effects upon disambiguation (e.g., Clifton Jr., 1993; Frazier & Rayner, 1982; Fujita & Cunnings, 2021a, 2021b; Jacob & Felser, 2016; Pickering & Traxler, 1998; Slattery et al., 2013; Sturt et al., 1999; Tabor & Hutchins, 2004). Garden-path effects in clause-boundary ambiguities suggest that the parser initially assigns the embedded object structure and subsequently has difficulty analysing the disambiguating input requiring the matrix subject DP.^3^

The parsing process after disambiguation (*the revision process*) has been the subject of much debate (e.g., Abney, 1989; J. D. Fodor & Ferreira, 1998; J. D. Fodor & Inoue, 1994; Gibson, 1991; Gorrell, 1995; Pritchett, 1992; Sturt, 1997).^4^ The debate is partly due to the finding that locally assigned misinterpretations persist after disambiguation (*lingering misinterpretation*; Christianson et al., 2001; Cunnings & Fujita, 2021; Fujita & Cunnings, 2020, 2021a, 2021b; Jacob & Felser, 2016; Sturt, 2007; van Gompel et al., 2006). For example, several studies have examined clause-boundary garden-path sentences, as exemplified in (1), and unambiguous sentences, such as *After Mary dressed, John bathed himself*. In this unambiguous sentence, the presence of a comma after the embedded verb prevents the parser from analysing “John” as the embedded object. After reading ambiguous or unambiguous sentences, participants in these studies answered questions referring to misinterpretation, for example, *Did Mary dress John?* for (1). The correct answer to this question is “no” because Mary dressed herself, not John. The studies have observed low comprehension accuracy when the comma is absent, suggesting that interpretations derived from the embedded object structure persist after disambiguation. Some studies have also argued or found that misinterpretations become more persistent, or revision becomes more difficult, when the locally ambiguous phrase is lengthened, for example, by a relative clause, as in *After Mary dressed the boy that was small bathed himself* (e.g., Crocker, 1996; Ferreira & Henderson, 1991; Fujita & Cunnings, 2020; Tabor & Hutchins, 2004).

Recent research has explored the online revision process by examining the syntactic structure assigned during revision (Fujita, 2021b; Fujita & Cunnings, 2021b; Slattery et al., 2013). Slattery et al. pioneered this line of research by testing sentences as follows.

- (3a) Before the friends left(,) [_TP_ Dean’s aunt arrived and introduced herself to the guests].
- (3b) Before the friends left(,) [_TP_ Dean’s uncle arrived and introduced herself to the guests].

In (3a/b), “Dean’s aunt/uncle” is locally ambiguous when the comma is absent. These sentences contain a reflexive pronoun, which is an expression without independent reference and referentially dependent on another DP. Structural constraints restrict co-reference to a *c-commanding* DP within the reflexive’s *binding domain*, the smallest XP with an intervening subject containing the reflexive (see Chomsky, 1981, 1986).^5^ C-command refers to a structural relation between nodes, defined as follows: x c-commands y if and only if x is a sister of y or x is a sister of z and z dominates y (see Reinhart, 1976).^6^ Binding is defined as follows: x binds y if and only if x c-commands y and they share the same index (Chomsky, 1981). According to the structural constraints, the reflexive in (3a/b) must co-vary with the locally ambiguous DP that either agrees (3a) or disagrees (3b) with it in gender ([_TP_ [_DPt_ Dean’s aunt/uncle] [_VP_ [_VP_ (t)race] and [_VP_ t [_DP_ herself]]]]; Burton & Grimshaw, 1992; Chomsky, 1981; Koopman & Sportiche, 1991; McNally, 1992; Sportiche, 1988; Woolford, 1991).^7^ There is considerable evidence that processing difficulty occurs at a pronoun when it and its structurally licensed antecedent disagree in gender (*gender mismatch effects*; see Cunnings & Sturt, 2014; Dillon et al., 2013; Fujita, 2021b, 2023; Fujita & Cunnings, 2021b; Giskes & Kush, 2021; Hall & Yoshida, 2021; Kazanina et al., 2007; Kush et al., 2017; Schneider & Phillips, 2001; Slattery et al., 2013; Sturt, 2003; Yoshida et al., 2013). Based on these studies, reading times should be longer at the reflexive in (3b) than in (3a) when the comma is present. In the comma-less conditions, if the locally ambiguous DP is analysed as the embedded object even after disambiguation, we can expect no gender mismatch effects due to the absence of a c-command relation with the reflexive ([_CP_ [_PP_ [_TP_ [_VP_ [_DPk_]]]] [_TP_ [_DPj_t_ ] [_VP_ t [_DPj/*k_ herself]]]]). Slattery et al. tested these hypotheses in a reading task and observed gender mismatch effects in both ambiguous and unambiguous sentences.

The results of Slattery et al. (2013) suggest that the parser analyses the locally ambiguous DP as the matrix subject after disambiguation. However, there are different hypotheses about the representations assigned during the revision process. Slattery et al. argue that the matrix subject DP and the embedded object DP coexist after disambiguation (e.g., [_CP_ [_PP_ [_TP_ [_VP_ [_DP1_ ]]]] [_TP_ [_DP2_ ] [_VP_ ]]] assigned to (3a/b), DP1 = DP2; see also J. D. Fodor & Inoue, 1998). Fujita (2021b) claims that the parser respects one-to-one correspondences between input strings and output representations. According to this claim, the locally ambiguous DP should function only as the matrix subject after disambiguation. It is also conceivable that the parser regards clause-boundary garden-path sentences as ungrammatical and analyses a nearby DP (*a local DP*) as the matrix subject (J. D. Fodor & Inoue, 1998, 2000; Meng & Bader, 2000). Janet Dean Fodor and Inoue (2000) argue that if disambiguating input provides a low degree of diagnosticity for disambiguation, the parser might dispense with the revision process to avoid wasting processing resources (*triage*). In this paper, I use the term “triage” to refer to the parser’s decision not to assign corresponding syntactic structures to input strings to prioritise other things (e.g., to conserve resources). In the language comprehension literature, analogous concepts have been proposed using various terms, including *good-enough representations*, *noisy-channel inference*, *processing overload* and *shallow parsing* (e.g., see Clahsen & Felser, 2006; Ferreira & Patson, 2007; J. A. Fodor, 1983; Gibson, 1991; Gibson et al., 2013; Sanford & Sturt, 2002). These terms are often associated with specific approaches to language comprehension that reflect different sources of incompatibility between input strings and output representations. I discuss some of these approaches in the *General discussion*. Note that triage, as the name implies, does not indicate the parser’s inability to assign syntactic structures compatible with input strings. Instead, it signifies a choice made by the parser to abstain from performing such assignments during the online parsing process. Janet Dean Fodor and Inoue (1998) propose that, when faced with clause-boundary ambiguities, the parser encounters significant difficulty identifying the matrix subject DP (e.g., see Bader, 1998; J. D. Fodor & Ferreira, 1998; Gibson, 1991; Sturt et al., 1999; Sturt & Crocker, 1998). Consequently, the parser analyses a local DP as the matrix subject (*the locality hypothesis*). This hypothesis builds on the well-established finding that the parser prefers to analyse an incoming element as part of the most local structure during sentence processing (e.g., Abney, 1989; L. Frazier, 1979; L. Frazier & J. D. Fodor, 1978; Gibson, 1991; Kimball, 1973; Phillips & Gibson, 1997). The locality hypothesis represents one potential parsing process after triage. In Slattery et al.’s experimental sentences, the locally ambiguous string is adjacent to the disambiguating verb. Their results are therefore compatible with the locality hypothesis. Fujita (2021b) recently tested this hypothesis in *complement ambiguities*, as in (4a/b).

- (4a) [_TP_ The nurses noticed [_CP_ (that) the mother of Maria visited the hospital to introduce herself to the doctor during lunch]].
- (4b) [_TP_ The nurses noticed [_CP_ (that) the father of Maria visited the hospital to introduce herself to the doctor during lunch]].

In (4a/b), “the mother/father of Maria” must be parsed as the embedded subject DP. However, in the absence of the overt complementiser following the matrix verb, this substring can be locally analysed as the matrix object DP, as in *[*_*TP*_ *The nurses [*_*VP*_ *noticed [*_*DP*_ *the mother/father of Maria]]]*. Studies have demonstrated parsing preferences for the matrix object analysis at the locally ambiguous region (e.g., Frazier & Rayner, 1982; Fujita, 2021b; Sturt et al., 1999). The sentences in (4a/b) contain a reflexive that either agrees or disagrees with the gender of its antecedent *PRO* that covaries with the locally ambiguous DP ([_TP_ [_DPj_ the mother/father of Maria] [_VP_ visited the hospital [_CP_ [_TP_ [_DPj_t_ PRO] to [_VP_ t introduce [_DPj_ herself]]]]]]).^8^ Covariance does not hold if the locally ambiguous DP is analysed as the matrix object (for theories on PRO, see Boeckx et al., 2010; Chomsky, 1981; Hornstein, 2003). One crucial difference from Slattery et al.’s research design is that the sentences in (4a/b) contain a local DP ([_DP_ Maria]), which does not referentially relate to the reflexive. However, “Maria” is adjacent to the disambiguating verb. The local DP matches the reflexive’s gender in all conditions. If the locality hypothesis holds for complement garden-path sentences, the parser should analyse only the local DP as the embedded subject upon disambiguation. Consequently, a co-reference relation should be established with it, which should lead to the absence of gender mismatch effects in ambiguous sentences. Contrary to this hypothesis, Fujita observed processing difficulty at the reflexive in gender-mismatch sentences, irrespective of the overtness of the complementiser. Fujita concludes that the parser revises complement garden-path sentences during the online parsing process.

The results of Fujita (2021b) are incompatible with the locality hypothesis. However, given that clause-boundary ambiguities may cause increased revision difficulty relative to complement ambiguities (e.g., Sturt et al., 1999), a different mechanism may underlie the online revision process in clause-boundary garden-path sentences. Indeed, Janet Dean Fodor and Inoue (1998) consider the differential difficulty and argue that the locality hypothesis does not hold for complement ambiguities.

In summary, studies have observed that the parser misanalyses clause-boundary garden-path sentences. However, the parsing process after disambiguation remains controversial. There are different hypotheses about the online revision process in clause-boundary ambiguities (J. D. Fodor & Inoue, 1998; Fujita, 2021b; Slattery et al., 2013), but existing empirical data do not disentangle these hypotheses. Furthermore, we know little about whether triage occurs and what might cause it in clause-boundary garden-path sentences. The present study investigated these issues using *relative clauses* (Bianchi, 2000; Chomsky, 1965, 1977, 1981; Citko, 2001; de Vries, 2002; Donati & Cecchetto, 2011; Douglas, 2016; Kayne, 1994; Ross, 1967; Safir, 1999; Schachter, 1973; Smith, 1964). In the following, before describing the research design employed, I briefly illustrate syntactic structures of relative clauses and discuss how they may be analysed during the online parsing process.

- (5a) The woman [_CP_ that visited Rebecca] dropped a wine bottle.
- (5b) The woman [_CP_ that Rebecca visited] dropped a wine bottle.

The sentences in (5a/b) contain a relative clause introduced by “that”. This relative clause modifies the noun “woman” (*the RC head*), which must function either as the subject (5a) or as the object (5b) within the relative clause. I refer to the relative clause in (5a) as *the subject relative clause* (*SRC*) and the one in (5b) as *the object relative clause* (*ORC*). Because of the relative clause, the RC head in (5a/b) has two functions: one in the relative clause and one outside of it. One explanation for this dual function is that the RC head raises from either the subject or object position to the matrix subject position via the specifier position of the CP (e.g., [_TP_ [_DP_ The [_NP_ [_NPt2_ woman] [_CP_ [_DPt1_ OP t2] that Rebecca visited t1]]]] dropped a wine bottle]).

The RC head and the traces form dependency relations. I refer to this process as *dependency formation* (for studies on the formation of dependencies, see Aoshima et al., 2004; Dillon et al., 2013; J. D. Fodor, 1978; Fujita & Cunnings, 2020, 2022, 2023; González Alonso et al., 2021; Hall & Yoshida, 2021; Jäger et al., 2017; Kazanina et al., 2007; Kim et al., 2020; Stowe, 1986; Wagers et al., 2009; Wagers & Phillips, 2014; Yoshida et al., 2014. For the definition of dependency relations adopted in this study, see footnote 4). In this study, I assume that the parser forms the dependencies in (5a/b) as follows. Upon recognising a relative clause, the parser assigns its entire syntactic structures, posits the traces and forms the dependencies. When encountering an actual trace in the subject or object position, the parser relates it with the corresponding (postulated) verb.

Some studies have reported that English ORCs cause increased processing difficulty relative to SRCs (e.g., Cunnings & Fujita, 2021a; Gibson, 1998; King & Just, 1991; Lau & Tanaka, 2021; Traxler et al., 2002; Warren & Gibson, 2002). One potential source of this difficulty is the so-called *ORC disadvantage*, which has been the subject of various approaches to language comprehension (e.g., Frazier, 1987; Gibson, 1998; King & Just, 1991; O’Grady, 1997). For example, *the resource-based approach* postulates that the ORC disadvantage results from an increased demand on memory resources in processing ORCs. This approach relies on the memory retention hypothesis that the longer the parser holds an element in memory, the more resources it would consume (e.g., see De Vincenzi, 1991; Gibson, 1998, 2000; Kim et al., 2020). As observed in (5a/b), the distance between the dependency entries is greater in ORCs than in SRCs. Also, another discourse referent appears during memory retention in ORCs. The resource-based approach assumes that these factors increase processing costs in ORCs (Gibson, 1998; King & Just, 1991). Others argue that the ORC disadvantage results from garden-path effects because when recognising a relative clause, the parser predictively constructs an SRC to minimise the distance between the dependency entries (e.g., De Vincenzi, 1991; Frazier, 1987). There are other approaches to the ORC disadvantage (see Lau & Tanaka, 2021), but existing theories, including those described above, assume that the cause of the ORC disadvantage lies inside the relative clause. Therefore, in this study, the ORC disadvantage refers to processing difficulty that occurs in the domain of a relative clause. This difficulty may affect the online revision process.

Crucially, several studies have reported that the ORC disadvantage in English does not occur or at least is attenuated when the RC head and the RC subject are dissimilar along a specific dimension (e.g., a definite description vs. a proper name; *[The woman] that [the girl] saw… vs. [The woman] that [Rebecca] saw…*; see Cunnings & Fujita, 2023; Gordon et al., 2001, 2006). This finding suggests that the similarity of DPs encoded during the parsing of relative clauses modulates or influences the ORC disadvantage. Despite the previous finding of this *similarity-based encoding interference*, Ferreira and Henderson (1998) reported that ORCs increase revision difficulty relative to SRCs in clause-boundary ambiguities when the two critical DPs are dissimilar. In their study, participants read clause-boundary garden-path sentences with either an SRC (6a) or an ORC (6b). Participants’ task was to judge their grammaticality in a rapid serial visual presentation.

- (6a) When the boy scratches the dog that hates Sally yawns loudly.
- (6b) When the boy scratches the dog that Sally hates yawns loudly.

Ferreira and Henderson observed higher grammaticality ratings in (6a) than (6b) but similar ratings in control versions of (6a/b), such as *When the boy scratches the dog that hates Sally/that Sally hates the girl yawns loudly*. These findings suggest that ORCs increase revision difficulty relative to SRCs, the source of which is irrelevant to similarity-based encoding interference.

With the previous findings reviewed above in mind, the present study conducted two self-paced reading experiments. Experiment 1 aimed to disentangle the existing hypotheses on the revisability of clause-boundary ambiguities (J. D. Fodor & Inoue, 1998; Fujita, 2021b; Slattery et al., 2013). To this end, Experiment 1 tested clause-boundary garden-path and unambiguous sentences with an SRC embedded within the matrix subject DP, as follows.

- (7) While the friends telephoned(,) the woman/gentleman that visited Rebecca cut herself on a piece of broken glass.

In (7), the SRC introduces a DP that is adjacent to the disambiguating verb in the surface form (i.e., a local DP) and that matches the reflexive in gender. The locality hypothesis predicts that the parser analyses only this DP as the matrix subject after disambiguation. Thus, according to this hypothesis, gender mismatch effects should be absent in ambiguous sentences. If the entire locally ambiguous DP is analysed as the matrix subject after disambiguation, gender mismatch effects should occur in ambiguous sentences, as observed in previous research (Fujita & Cunnings, 2021b; Slattery et al., 2013).

Experiment 2 aimed to investigate what increases revision difficulty and whether it leads to triage in clause-boundary ambiguities. For this investigation, Experiment 2 tested ambiguous and unambiguous sentences, as in Experiment 1, but with an ORC, as follows.

- (8) While the friends telephoned(,) the woman/gentleman that Rebecca visited cut herself on a piece of broken glass.

The ORC in (8) introduces a local DP, which is a proper name, as opposed to the RC head, which is a definite description, as in (7). As described earlier, the English language may be subject to the ORC disadvantage (King & Just, 1991), but it may not occur when the RC-head and the RC subject are dissimilar, as in (8) (Cunnings & Fujita, 2023; Gordon et al., 2006). Nevertheless, ORCs may lead to increased revision difficulty in clause-boundary ambiguities relative to SRCs (Ferreira & Henderson, 1998), suggesting that there is a factor, irrelevant to similarity-based encoding interference, that affects revision difficulty. If this factor, along with garden-path effects, leads to triage, gender mismatch effects should be absent in (8) when the comma is absent.

A brief overview of the results: Experiments 1 and 2 showed garden-path effects at the disambiguating region, suggesting that the parser misanalyses clause-boundary garden-path sentences. In Experiment 1, gender mismatch effects were observed at the post-reflexive region in both ambiguous and unambiguous sentences. However, in Experiment 2, gender mismatch effects were present only in unambiguous sentences. Experiment 2 also showed longer reading times at the reflexive in ambiguous than unambiguous sentences when the locally ambiguous DP matched the reflexive in gender. The observations at the (post-)reflexive regions suggest that the parser revises clause-boundary garden-path sentences after disambiguation (Experiment 1), but triage occurs when an ORC is embedded within the locally ambiguous DP (Experiment 2). Ambiguity effects observed at the reflexive in Experiment 2 indicate that the parser attempts to resolve a reflexive immediately after encountering it but has difficulty doing so. This finding suggests that no element occupies the matrix subject position after disambiguation when an ORC is present, which is consistent with the absence of gender mismatch effects. It also suggests that, after triage at the disambiguating region, the parser continues to analyse the matrix clause rather than abandoning the analysis of it.

## Method

### Experiment 1

Experiment 1 investigated the revision process in clause-boundary garden-path sentences, as below.

- (9a) *Ambiguous*, *gender match*

While the friends telephoned the woman that visited Rebecca dropped a wine bottle and cut herself on a piece of broken glass.

- (9b) *Ambiguous*, *gender mismatch*

While the friends telephoned the gentleman that visited Rebecca dropped a wine bottle and cut herself on a piece of broken glass.

- (9c) *Unambiguous*, *gender match*

While the friends telephoned, the woman that visited Rebecca dropped a wine bottle and cut herself on a piece of broken glass.

- (9d) *Unambiguous*, *gender mismatch*

While the friends telephoned, the gentleman that visited Rebecca dropped a wine bottle and cut herself on a piece of broken glass.

Regions: While the friends | telephoned(,) | the woman/gentleman | that visited Rebecca | dropped | a wine bottle | and cut | herself | on a piece of | broken glass.|

The substring “the gentleman/woman that visited Rebecca” is locally ambiguous in (9a/b) but not in (9c/d) because of a comma. The sentences in (9a–d) contain a reflexive that either matches (9a/c) or mismatches (9b/d) its structurally licensed antecedent (i.e., the locally ambiguous DP) in gender. Crucially, co-reference between these DPs does not hold if the locally ambiguous DP is analysed as the embedded object. The sentences in (9a–d) also contain another DP ([_DP_ Rebecca]), which does not c-command the reflexive but is adjacent to the disambiguating region in the surface form. This local DP matches the reflexive’s gender in all conditions.

If the parser misanalyses the locally ambiguous DP as the embedded object (e.g., Frazier & Rayner, 1982), garden-path effects should occur at the disambiguating region in (9a/b). Also, if binding constraints (Chomsky, 1981) apply during the online parsing process, reading times should be longer at the reflexive in (9d) than (9c). The crucial question is whether this gender mismatch effect also occurs in ambiguous sentences. If the parser analyses the locally ambiguous DP as the matrix subject after disambiguation (Fujita & Cunnings, 2021b; Slattery et al., 2013), gender mismatch effects should be present in ambiguous sentences as well. If the parser analyses only the local DP as the matrix subject (J. D. Fodor & Inoue, 1998), gender mismatch effects should be absent. If triage ensues after disambiguation but the locality hypothesis does not hold, there are two possible consequences. One is that the locally ambiguous DP remains in the embedded object position after disambiguation, and the parser analyses the matrix clause as devoid of an element in the subject position. In this case, in addition to the absence of gender mismatch effects, reading times at the reflexive should be longer in (9a) than (9c) because the parser should have difficulty resolving the reflexive. Alternatively, the parser may give up on analysing the matrix clause after disambiguation. In this case, reading times at the reflexive should be similar between (9a) and (9c), or shorter in (9a) than (9c), because the parser does not need to establish co-reference relations.

### Participants

In Experiment 1, 151 native English speakers were recruited via Prolific (https://www.prolific.co). These participants completed Experiment 1 online. Before data analysis, I excluded the data of 11 participants due to their low comprehension accuracy (< 70%). The participants included in data analysis (*N* = 140) were university students, aged between 18 and 40 years, monolingual English speakers and were British citizens.

### Materials

Materials were 24 sets of experimental sentences, as in (9a–d), and 72 filler sentences. A yes/no comprehension question followed all experimental and two-thirds of the filler sentences. Comprehension questions for experimental sentences did not query local ambiguity or the reflexive’s antecedent.^9^ The experimental sentences are available via the Open Science Framework at https://osf.io/78vmz/.

### Procedure

In Experiment 1, a non-cumulative phrase-by-phrase self-paced reading task, created using code available online (Fujita, 2021a), was administered in IbexFarm to measure participants’ reading times. In this task, participants read each phrase by pressing the space bar. When they finished reading the last phrase, the sentence disappeared, and either the next trial or a comprehension question appeared. Participants answered each question by pressing either the ‘1’ or ‘2’ key. The experiment began with four practice trials.

### Data analysis

For data analysis, linear mixed-effects models were fitted with full variance-covariance matrices for the random effects (*the maximal model*) using the lme4 package (Bates et al., 2015) in R (R Core Team, 2020). The dependent variable was log-transformed reading times at the (post-)disambiguating (“dropped”/“a wine bottle”) and (post-)reflexive (“herself”/“on a piece of”) regions. Before data analysis, reading times shorter than 200 ms or longer than 6,000 ms were excluded, which represented less than 0.01 % of the data.^10^ Fixed effects were sum-coded (.5/–.5) main effects of ambiguity (ambiguous/unambiguous) and gender (match/mismatch), and their interactions. When the maximal model did not converge, random effects correlations were initially removed. The model was then simplified by iteratively removing the random effects accounting for the least variance until it converged. To analyse the data, *p* values were estimated from the *t* distribution (Baayen, 2008), and those less than .05 were interpreted as significant. Data and analysis code are available via the Open Science Framework at https://osf.io/78vmz/.

### Results

Average comprehension accuracy of the experimental and filler materials was 87% (range 72–98). Table 1 summarises inferential statistics, and Fig. 2 illustrates reading times at the regions of theoretical interest.^11^

**Table 1:** Inferential statistics in Experiment 1

	Disambiguating region				Post-disambiguating region			
	Estimate	SE	*t*	*p*	Estimate	SE	*t*	*p*
Intercept	6.349	0.03	229.61	< .001	6.296	0.03	230.13	< .001
Ambiguity	0.058	0.02	3.44	< .001	0.068	0.01	4.73	< .001
Gender	0.004	0.01	0.28	.779	0.028	0.01	1.94	.052
Ambiguity × Gender	0.005	0.03	0.19	.849	0.005	0.03	0.18	.858
	Reflexive region				Post-reflexive region			
	Estimate	SE	*t*	*p*	Estimate	SE	*t*	*p*
Intercept	6.075	0.02	301.00	< .001	6.118	0.03	200.40	< .001
Ambiguity	0.010	0.01	0.97	.334	–0.011	0.01	–0.93	.354
Gender	0.019	0.01	1.94	.052	0.036	0.01	3.06	.002
Ambiguity × Gender	–0.016	0.02	–0.93	.350	–0.001	0.02	–0.05	.961

#### Disambiguating and post-disambiguating regions

Analysis showed a significant main effect of ambiguity at the disambiguating region, with longer reading times in the ambiguous than unambiguous conditions. This garden-path effect was also present at the post-disambiguating region.

#### Reflexive and post-reflexive regions

There were no statistically significant effects at the reflexive region. At the post-reflexive region, the main effect of gender was statistically significant, showing longer reading times in the gender-mismatch than gender-match conditions. The ambiguity by gender interaction was not statistically significant.

### Discussion

The results showed garden-path effects at the (post-)disambiguating regions, suggesting that the parser misanalyses the locally ambiguous DP. Crucially, gender mismatch effects were present at the post-reflexive region in the absence of an interaction with ambiguity. These findings suggest that the parser revises the locally ambiguous DP as the matrix subject after disambiguation.

Experiment 1 provided evidence against the locality hypothesis and suggested that, in clause-boundary garden-path sentences, the parser assigns grammatical structures corresponding to the disambiguating input. Experiment 2 investigated the revision process using ORCs. As discussed in the *Introduction*, ORCs in English may incur additional processing costs compared to SRCs (King & Just, 1991), and this ORC disadvantage may be due to the similarity of the RC head and the RC subject (Cunnings & Fujita, 2023; Gordon et al., 2006). Nevertheless, in a grammaticality judgement task, Ferreira and Henderson (1998) observed that an ORC embedded within the locally ambiguous DP led to an increased number of “ungrammatical” responses in clause-boundary garden-path sentences relative to an embedded SRC when the two DPs were dissimilar. This observation suggests that ORCs introduce at least one factor that is irrelevant to similarity-based encoding interference that increases revision difficulty. Experiment 2 tested whether this increased difficulty affects the revision process.

### Experiment 2

Experiment 2 investigated whether ORCs affect the online revision process, as below.

- (10a) *Ambiguous*, *gender match*

While the friends telephoned the woman that Rebecca visited dropped a wine bottle and cut herself on a piece of broken glass.

- (10b) *Ambiguous*, *gender mismatch*

While the friends telephoned the gentleman that Rebecca visited dropped a wine bottle and cut herself on a piece of broken glass.

- (10c) *Unambiguous*, *gender match*

While the friends telephoned, the woman that Rebecca visited dropped a wine bottle and cut herself on a piece of broken glass.

(10d) *Unambiguous*, *gender mismatch*

While the friends telephoned, the gentleman that Rebecca visited dropped a wine bottle and cut herself on a piece of broken glass.

Regions: While the friends | telephoned(,) | the woman/gentleman | that Rebecca visited | dropped | a wine bottle | and cut | herself | on a piece of | broken glass.|

The sentences in (10a–d) have an ORC embedded within the matrix subject DP. The RC head is a definite description, whereas the RC subject is a proper name. Thus, there should be no similarity-based encoding interference. If the ORC still increases processing costs and leads to triage, gender mismatch effects should be absent in (10b). There may also be ambiguity effects at the reflexive in (10a) compared to (10c), depending on how the parser analyses the matrix clause after disambiguation. If ORCs do not affect the revision process, the results should be akin to those obtained in Experiment 1.

#### Participants

In Experiment 2, 147 native English speakers who did not participate in Experiment 1 were recruited via Prolific and completed the task online. Data from seven participants were excluded due to low comprehension accuracy (< 70%). Thus, data analysis included 140 participants. The participant pool was the same as in Experiment 1.

#### Materials

Experiment 2 contained 24 sets of experimental sentences, as in (10a–d), and 72 filler sentences. As in Experiment 1, a yes/no question followed all experimental sentences and two-thirds of the filler sentences, and comprehension questions for experimental sentences did not probe local ambiguity or the reflexive’s antecedent.

#### Procedure and data analysis

The procedure and data analysis were identical to those of Experiment 1.

### Results

Average comprehension accuracy of the experimental and filler materials was 87% (range 71–98). Table 2. reports inferential statistics, and Figure 3 illustrates reading times at the (post-)disambiguating and (post-)reflexive regions.^12^

**Table 2:** Inferential statistics in Experiment 2

	Disambiguating region				Post-disambiguating region			
	Estimate	SE	*t*	*p*	Estimate	SE	*t*	*p*
Intercept	6.409	0.03	215.79	< .001	6.307	0.03	232.28	< .001
Ambiguity	0.070	0.02	4.32	< .001	0.077	0.02	4.37	< .001
Gender	–0.010	0.02	–0.63	.530	0.012	0.02	0.78	.433
Ambiguity × Gender	–0.019	0.03	–0.65	.513	–0.011	0.03	–0.38	.704
	Reflexive region				Post-reflexive region			
	Estimate	SE	*t*	*p*	Estimate	SE	*t*	*p*
Intercept	6.082	0.02	306.99	< .001	6.141	0.03	212.65	< .001
Ambiguity	0.001	0.01	0.13	.900	–0.034	0.01	–2.64	.008
Gender	0.019	0.01	1.85	.064	0.039	0.01	2.91	.004
Ambiguity × Gender	–0.055	0.02	–2.83	.005	–0.068	0.02	–3.01	.003
First nested model								
Gender: ambiguous conditions	–0.008	0.01	–0.62	.537	0.005	0.02	0.33	.743
Gender: unambiguous conditions	0.047	0.02	3.11	.002	0.073	0.02	4.25	< .001
Second nested model								
Ambiguity: gender-match conditions	–0.029	0.01	–2.23	.026	0.000	0.02	0.02	.987
Ambiguity: gender-mismatch conditions	0.026	0.02	1.67	.095	0.068	0.02	3.89	< .001

#### Disambiguating and post-disambiguating regions

There was a significant main effect of ambiguity at the disambiguating region, with longer reading times in the ambiguous than unambiguous conditions, suggesting garden-path effects. The post-disambiguating region also showed garden-path effects.

#### Reflexive and post-reflexive regions

There was a significant main effect of gender only at the post-reflexive region. Crucially, the interaction between ambiguity and gender was statistically significant at the (post-)reflexive regions. Two nested models were fitted to explore these interactions. The first model examined the effect of gender by sum-coding it within each level of ambiguity for each region. This model showed gender mismatch effects in the unambiguous conditions but not in the ambiguous conditions for both regions. The second model examined ambiguity effects within each level of gender for each region. This model showed significant ambiguity effects at the reflexive in the gender-match conditions.

### Discussion

Consistent with Experiment 1, garden-path effects were observed at the (post-)disambiguating regions, suggesting misanalysis of the locally ambiguous DP. Crucially, gender mismatch effects were present at the (post-)reflexive regions only in the unambiguous conditions. The absence of gender mismatch effects in the ambiguous conditions suggests that the parser does not revise the locally ambiguous DP as the matrix subject. There was also evidence of ambiguity effects at the reflexive in the gender-match condition, indicating that the parser attempts to resolve a reflexive upon encountering it but has difficulty doing so. This observation suggests that the parser continues to analyse sentences after disambiguation and that no element occupies the matrix subject position at the point of the reflexive, the latter supporting the finding that the parser does not complete the revision process.

## General discussion

The present study investigated the parsing of clause-boundary garden-path sentences in two self-paced reading experiments. Recall that this study had two aims. One was to investigate the revisability of clause-boundary garden-path sentences. The other was to explore whether ORCs affect the revision process in the absence of similarity-based encoding interference.

Experiments 1 and 2 showed garden-path effects at the disambiguating region, aligning with extensive research indicating that the parser misanalyses clause-boundary garden-path sentences (e.g., Frazier & Rayner, 1982). Crucially, the two experiments revealed different parsing processes after disambiguation. In Experiment 1, the results suggested that the parser revises clause-boundary garden-path sentences, as evidenced by the presence of gender mismatch effects in ambiguous sentences, whereas in Experiment 2, there was evidence that it does not, as demonstrated by the absence of gender mismatch effects. Experiment 2 also showed increased processing difficulty at the reflexive in ambiguous relative to unambiguous sentences when the reflexive and its antecedent matched in gender. This ambiguity effect suggests that the parser attempts to resolve a reflexive upon encountering it but has difficulty doing so. Below, I discuss the implications of these results for the two aims in turn.

### Revision in clause-boundary garden-path sentences

Regarding the first aim, as described in the *Introduction*, some claim that the parser analyses the locally ambiguous DP as the matrix subject after disambiguation (Fujita, 2021b; Slattery et al., 2013), whereas others argue that the parser analyses a local DP as the matrix subject (J. D. Fodor & Inoue, 1998). Experiment 1 presented evidence against the locality hypothesis and suggested that the parser assigns grammatical structures corresponding to the disambiguating input during the online parsing process.

It is worth noting that the experimental sentences tested in Experiment 1 are extremely difficult to parse. The difficulty is not only because clause-boundary ambiguities may lead to a high degree of revision difficulty (e.g., Sturt et al., 1999), but also because a relative clause lengthens the locally ambiguous DP. As noted in the *Introduction*, there is evidence that revision difficulty increases as the parser becomes more committed to misanalysis (e.g., Ferreira & Henderson, 1991; Fujita & Cunnings, 2020; Tabor & Hutchins, 2004). Nevertheless, Experiment 1 showed that the parser revises the locally ambiguous DP as the matrix subject. This finding suggests that the human parser is capable of analysing highly complex syntactic structures during online sentence processing.

Experiment 1 also provides insight into the source of lingering misinterpretation in garden-path sentences. As noted in the *Introduction*, the debate about the revisability of clause-boundary ambiguities has been driven in part by the finding that locally assigned misinterpretations linger after disambiguation (e.g., Fujita & Cunnings, 2021b; Jacob & Felser, 2016). Crucially, some of these studies have observed lingering misinterpretation when garden-path sentences are potentially easier to revise than those tested in Experiment 1, for example, due to the absence of a relative clause (e.g., Jacob & Felser, 2016). Experiment 1 suggests that lingering misinterpretation observed in these studies is not due to a failure to revise the locally ambiguous DP as the matrix subject.

The results on the revisability of clause-boundary garden-path sentences also have implications for online reflexive resolution. As described in the *Introduction*, structural constraints regulate co-reference relations (Chomsky, 1981), and the parser obeys these constraints during the online parsing process. Previous research has provided substantial evidence for the online application of structural constraints through the observation of processing difficulty due to gender incongruence between reflexives and their structurally licensed antecedents. Building on this well-established phenomenon, the present study investigated the revision process, and Experiment 1 showed gender mismatch effects in ambiguous sentences while Experiment 2 did not. Although the following is circular reasoning, we can take these results as novel evidence that online reflexive resolution obeys structural constraints. That is, gender mismatch effects occur in clause-boundary garden-path sentences with an SRC because the locally ambiguous DP functions as the matrix subject after disambiguation. When an ORC is present, gender mismatch effects do not occur because the locally ambiguous DP does not occupy the matrix subject position. Furthermore, Experiment 2 showed ambiguity effects at the reflexive, providing additional evidence that the parser searches for the structurally licensed position for reflexive resolution. Again, the discussion here revolves in a circular manner, given the rationale underlying the research design employed in this study. However, Experiments 1 and 2 align with the growing body of evidence that online reflexive resolution is a structure-dependent process (e.g., see Dillon et al., 2013; Fujita & Yoshida, 2023; Sturt, 2003).

### The triage process

Regarding the second aim, Experiment 2 provided evidence that the parser does not revise the locally ambiguous DP as the matrix subject. This finding suggests that triage is an option during the online parsing process. The cause of triage should pertain to ORCs, given that the only difference between Experiments 1 and 2 is the clausal modifier. In the following, I discuss why ORCs affect the revision process.

Janet Dean Fodor and Inoue (2000) discuss the scope of triage to formulate hypotheses about the mechanism underlying triage. Although they propose various hypotheses, their theoretical framework relies on the degree of diagnosticity of disambiguating cues. According to this *diagnostic approach* (J. D. Fodor & Inoue, 1998), some disambiguating cues are more diagnostic than others (e.g., see J. D. Fodor & Inoue, 1998; Fujita & Cunnings, 2020; Martin & McElree, 2018; Meng & Bader, 2000; Omaki et al., 2014), and the parser performs triage when a disambiguating cue is not sufficiently diagnostic. This approach would have difficulty accounting for the present study because the difference in relative clause types between Experiments 1 and 2 is irrelevant to the disambiguating verb and thus unlikely to influence the degree of diagnosticity.

Experiment 2 also provides evidence against the locality hypothesis with respect to the analysis of a local DP. Recall that the locality hypothesis makes two predictions: the parser does not complete the revision process and analyses a local DP as the matrix subject. As discussed earlier, the results of Experiment 1 contradict the first prediction. Regarding the second prediction, Experiment 2 showed that the parser has difficulty resolving a reflexive in ambiguous sentences. If the parser analysed only the local DP as the matrix subject after disambiguation, this difficulty should not occur. Thus, the results of Experiment 2 refute the second prediction of the locality hypothesis.

As discussed earlier, ORCs may incur higher processing costs than SRCs in English, which may affect the revision process (e.g., Cunnings & Fujita, 2023; Gibson, 1998; King & Just, 1991; Lau & Tanaka, 2021; Traxler et al., 2002; Warren & Gibson, 2002). However, it is known that this ORC disadvantage does not occur or at least is attenuated when the RC head and the RC subject are dissimilar (e.g., Cunnings & Fujita, 2023; Gordon et al., 2006), suggesting that similarity-based encoding interference is the main source of the ORC disadvantage. Nevertheless, Experiment 2, where the two DPs were dissimilar, presented evidence of triage. This finding is compatible with Ferreira and Henderson (1998), who reported increased revision difficulty with an ORC in an offline grammaticality judgement task, the cause of which was independent of similarity-based encoding interference. Why do ORCs increase revision difficulty in clause-boundary garden-path sentences? One possible answer is that the ORC disadvantage is not entirely attributable to similarity-based encoding interference, and it imposes additional processing costs at the disambiguating region.

The ORC disadvantage is a possible contributor to triage. However, given the evidence that similarity-based encoding interference modulates the ORC disadvantage, we need to consider other potential causes. There is also some evidence from the temporal aspect that the ORC disadvantage, if present, may not have influenced the revision process in Experiment 2. As noted in the *Introduction*, the ORC disadvantage occurs at the RC subject and/or verb. In the present study, these elements precede the disambiguating region (e.g., “While the friends telephoned the woman [that Rebecca visited] dropped…”). Therefore, if the ORC disadvantage occurs, we must assume that its effect spills over into the disambiguating region and that the two sources of processing costs lead to triage. However, some research has shown that the ORC disadvantage is an ephemeral phenomenon. For example, Staub et al. (2017) reported that when a prepositional phrase followed the RC verb, as in *The woman that the man visited before lunch dropped a wine bottle*, the ORC disadvantage observed at the RC subject was absent at the prepositional phrase and the matrix verb (but see Lowder & Gordon, 2021). This finding suggests that the ORC disadvantage is transient and therefore unlikely to spill over into the disambiguating region.

One possible factor that may have contributed to increased revision difficulty in Experiment 2 is the sequence of two verbs that appear across the relative clause and the disambiguating region (e.g., “While the friends telephoned the woman [that Rebecca [visited]] [dropped]…”; Ferreira & Henderson, 1998; Grodner & Gibson, 2005; Staub et al., 2017). As mentioned earlier, Staub et al. (2017) reported that the ORC disadvantage did not spill over. However, they observed processing difficulty at the matrix verb when no material followed the relative clause verb, as in *The woman that the man visited dropped a wine bottle* (see also Gordon et al., 2006; Traxler et al., 2002). The sequence of two verbs also appears in the materials of Ferreira and Henderson (1998), who reported that ORCs increase revision difficulty in clause-boundary ambiguities.

Why may consecutive verbs cause difficulty? Staub et al. (2017) argue that it is due to successive memory retrievals. To understand their argument, consider the online processing of the following ORC sentence, *The woman that the man visited dropped a wine bottle*. When the RC verb appears, the representation of the head RC is retrieved from memory to be analysed as the theme of the verb. The parser then encounters the matrix verb, where the representation of the matrix subject DP is retrieved for establishing a thematic relation with it. Thus, the parser engages in two successive memory retrievals of the corresponding entity over the relative clause and the matrix clause. According to Staub et al., these successive retrievals are difficult and occur serially. Thus, the second retrieval must await the completion of the first one. Because memory retrieval at the RC verb is assumed to be difficult, Staub et al. argue that it takes time and continues at the matrix verb. Staub et al. suggest that multiple retrievals at a single region cause processing difficulty.

Crucially, the hypothesis proposed by Staub et al. (2017) relies on the ORC disadvantage, as they predict retrieval difficulty at the RC verb. However, as discussed earlier, it is not clear whether the ORC disadvantage occurs in the materials tested in Experiment 2. Also, Staub et al.’s hypothesis does not align well with research that demonstrates or argues for the activation and ease of retrieval of an element that has been previously accessed in memory (e.g., Gibson & Warren, 2004; Keine, 2020).

One comprehensive hypothesis that may account for both the previous studies and the present one relates to shifts in the grammatical functions of the RC head during the online parsing process (MacWhinney & Pléh, 1988; Sheldon, 1974; Staub et al., 2017; Yngve, 1960). According to *the parallel grammatical function hypothesis*, processing difficulty occurs when the parser successively assigns different grammatical functions to referentially related elements. For example, consider the materials tested in Staub et al. (2017) again. In their ORC sentences (e.g., The woman that the man visited dropped a wine bottle), when the RC verb appears, the parser analyses the trace referentially related to the RC head as the object. However, at the matrix verb, the parser must analyse the matrix subject DP as the subject. Thus, Staub et al.’s ORC sentences require successive analysis of referentially related DPs as having different grammatical functions. According to the parallel grammatical function hypothesis, this incremental shift in grammatical functions causes processing difficulty. In SRC sentences (e.g., The woman that visited the man dropped a wine bottle), the relevant DPs’ grammatical functions remain unchanged between the regions of the RC object and the matrix verb. Thus, the parallel grammatical function hypothesis predicts no difficulty at the matrix verb. The same applies to the materials of the present study. In Experiment 1 (e.g., While the friends telephoned the woman that visited Rebecca dropped…), the two DPs’ grammatical functions do not change between the RC object and the matrix verb. In Experiment 2 (e.g., While the friends telephoned the woman that Rebecca visited dropped…), the parser must analyse the relevant DPs as having different grammatical functions across the RC verb and the matrix verb, and these analyses occur successively. Hence, the parallel grammatical function hypothesis predicts processing difficulty independent of garden-path effects at the disambiguating region. Assuming that the parallel grammatical function hypothesis holds, we can hypothesise that triage occurs as follows. When the disambiguating verb appears, garden-path effects arise. The parser then searches for the matrix subject DP and attempts to analyse the locally ambiguous DP as the subject. However, this attempt requires analysing the referentially related DPs as having different grammatical functions successively, causing additional difficulty. Consequently, the parser rejects this analysis and leaves the locally ambiguous DP in the embedded clause. Here, I assume that, although the locally ambiguous DP is considered as the matrix subject, the parser ultimately opts to dismiss this analysis. The driving force of this decision may pertain to the revision as a last resort hypothesis or some minimal cost principle (e.g., see De Vincenzi, 1991; J. D. Fodor & Frazier, 1980; J. D. Fodor & Inoue, 2000; Fujita, 2023).

The parallel grammatical function hypothesis provides a generalised account of the previous studies examining the parsing of relative clauses and the present study investigating the revision process. In what follows, I consider a hypothesis based on how the parser may perform revision when backtracking to preceding strings is unavailable, as in a self-paced reading task. There is a long-standing debate in the literature about revision strategies (e.g., Frazier & Rayner, 1982; Lewis, 1998; Meseguer et al., 2002; von der Malsburg & Vasishth, 2011, 2013). For example, some argue that readers’ gaze selectively returns to a locally ambiguous string during revision (Frazier & Rayner, 1982; Meseguer et al., 2002), while others claim that readers move their gaze back to the beginning of the sentence to reread (von der Malsburg & Vasishth, 2011, 2013). In both strategies, revision may involve backtracking to a previous state in the parse and selecting an alternative analysis (Sturt, 1997; Winograd, 1983; but see Fodor & Inoue, 1998). In a non-cumulative reading task, the revision process proceeds covertly (Frazier & Rayner, 1982; Lewis, 1998), retrieving a previous state from memory without regressive eye movements.

Although, to my knowledge, no research has experimentally investigated the underlying mechanism of the covert revision process, it is unlikely that participants in the present study engaged in forward revision. The reason is that this strategy imposes heavy demands on cognitive resources and therefore is time-consuming in a non-cumulative self-paced reading task (but not in a task where rereading is possible, as in an eye-movement-during-reading task; see Lewis, 1998); however, reading times at the disambiguating region were not notably long. Also, forward revision does not accord with triage. For example, upon disambiguation in clause-boundary garden-path sentences, forward revision entails the following process: the parser retrieves all lexical items preceding the disambiguating region, analyses the sentence from the beginning, locates the locally ambiguous DP and analyses it as the matrix subject. This revision process would take some time, as the parser must retrieve many lexical items upon disambiguation and analyse the sentence from the beginning. However, participants in Experiments 1 and 2 spent only 700–750 ms on average at the disambiguation region. Given such short reading times, it is unlikely that they engaged in forward revision.

In contrast, selective revision is a more plausible strategy because it would impose less cognitive load in a non-cumulative self-paced reading task (e.g., this strategy does not require retrieving all preceding lexical items upon disambiguation and analysing the sentence from the beginning). For selective revision, the parser needs information to locate the missing element (Frazier & Rayner, 1982; Sturt, 1997). In clause-boundary ambiguities, grammatical constraints provide two pieces of information at the disambiguating verb. Firstly, the missing element is a DP. Secondly, it has nominative Case. One hypothesis that we can formulate from the present study is that the parser gives significant weight to grammatical case during the revision of clause-boundary garden-path sentences.^13^ That is, if there is a DP that appears to have nominative Case during revision, the parser eagerly analyses it as the matrix subject (here, I assume that the parser searches only in a local region due to some locality constraint and that the temporal adjunct subject DP is outside this region). If this analysis is grammatically impermissible, the parser confronts a dilemma between its inclination to analyse a nominative Case assigned DP as the matrix subject and the grammatical constraints that disallow it. Consequently, triage occurs; the parser analyses the matrix clause as lacking an element in the subject position and proceeds to analyse the rest of the sentence. Note that DPs that appear in the materials of the present study bear Case in an abstract sense, determined by structural configurations. Therefore, the parser must rely on structural information to identify grammatical case. If there do not appear to be any DPs assigned nominative Case in the local region, the parser searches for the matrix subject without persisting with any ungrammatical analyses.^14^ When a DP is located, the parser checks that it is separable from its licensor (e.g., its Case assigner (or governor) or theta-role assigner; see Chomsky, 1981) and that it matches the matrix verb in phi-features (e.g., number). After these syntactic properties are checked, if the resultant structure is grammatical, the revision process is complete.

In Experiment 2, the local DP has nominative Case. Thus, the parser eagerly analyses it as the matrix subject. However, this analysis violates grammatical constraints (e.g., the relative clause loses a subject DP, which violates the EPP; Chomsky, 1982). Therefore, the parser does not adopt this analysis. However, it persists with it due to its inclination to analyse a nominative Case assigned DP as the matrix subject. Consequently, triage occurs. In the case that no DPs in the local region appear to have nominative Case, as in Experiment 1, the parser reaches the locally ambiguous DP at some point during revision. Since analysing this DP as the matrix subject does not violate any grammatical constraints, this analysis succeeds, and revision is complete.

The revision hypothesis outlined above merely describes the parser’s possible behaviour in clause-boundary garden-path sentences and does not explain the underlying mechanism. Also, several theoretical aspects of the online revision process remain unspecified, including the algorithm employed by the parser to search for DPs and the distance at which a DP must be located from the disambiguating region to fall outside the search range. Nevertheless, there is supportive evidence that the parser gives weight to grammatical case during revision. Meng and Bader (2000) conducted a grammaticality judgement task to investigate how case marking and number agreement as disambiguating cues influence the revision process of German garden-path sentences. Meng and Bader observed higher grammaticality ratings when the disambiguating cue was case marking compared to when it was number agreement. These results suggest that grammatical case plays a vital role in the revision process, aligning with the revision hypothesis delineated above.

### Implications for language comprehension approaches

Lastly, I discuss the implications for two approaches to language comprehension that consider the processing of garden-path sentences: *the good-enough language comprehension approach* (e.g., Ferreira et al., 2002; Ferreira & Patson, 2007; Slattery et al., 2013) and *the noisy-channel approach* (e.g., Futrell & Gibson, 2017; Gibson et al., 2013; Levy et al., 2009). Th good-enough approach views language comprehension as a process of understanding intended messages quickly and efficiently. Because such a process can be shallow (J. A. Fodor, 1983) and may not necessarily demand precise representations of input, the good-enough approach predicts that inaccurate representations are built during online language comprehension unless required by the task at hand. For clause-boundary ambiguities, the good-enough approach argues that, although the locally ambiguous DP is analysed as the matrix subject after disambiguation, it remains in the embedded object position as well (Slattery et al., 2013). This argument partially conflicts with the results of the present study because Experiment 2 demonstrated that the locally ambiguous DP does not occupy the matrix subject position after disambiguation. To reconcile with these results, the good-enough approach may need to assume that different types of clausal modifiers affect the revision process in distinct ways and that self-paced reading does not require a sufficiently high level of attention for the parser to revise the locally ambiguous DP containing an ORC as the matrix subject. If the good-enough approach adopts this perspective and positions itself as a linguistic (cognitive) theory (Chomsky, 1965), it needs to specify the mechanism underlying the online revision process and provide an exploratory account of why ORCs incur greater revision costs than SRCs.

The noisy-channel approach assumes that inferencing plays a crucial role in online language comprehension. Concretely, this approach argues that when readers recognise that the literal interpretation of a sentence is implausible or that the underlying syntactic structure is ill-formed, they infer the intended meaning and correct the sentence. Futrell and Gibson (2017) suggest that inferencing may be the source of lingering misinterpretation. They propose that, in clause-boundary garden-path sentences, such as *While the friends telephoned the woman dropped a wine bottle*, readers infer that a comma and the matrix subject are missing and correct them, such as *While the friends telephoned the woman, it dropped a wine bottle*. This proposal is partly inconsistent with the results of the present study because it incorrectly predicts no gender mismatch effects at the reflexive in Experiment 1 (e.g., a corrected string: *While the friends telephoned the woman/gentleman that visited Rebecca, it dropped a wine bottle and cut herself*). One way to reconcile with the results of the present study is to assume that readers make an inference when an ORC is embedded within the locally ambiguous DP but make a literal interpretation when an SRC is embedded. This assumption would be compatible with the noisy-channel approach if, for example, it assumes that triage in Experiment 2 results from increased processing costs and interprets processing costs as the level of noise. In this case, however, it is unclear how inferencing can become the cause of lingering misinterpretation when processing costs are low (e.g., Fujita & Cunnings, 2020, 2021a; Jacob & Felser, 2016; Sturt, 2007). Experiments 1 and 2 suggest that it is difficult to describe how readers comprehend locally ambiguous sentences based on inferencing alone.

## Conclusion

The present study investigated the online revision process in clause-boundary garden-path sentences through two self-paced reading experiments. These experiments demonstrated that the parser revises the locally ambiguous DP as the matrix subject when an SRC is embedded within it but not when an ORC is embedded. These findings suggest that the parser assigns grammatical structures corresponding to the disambiguating input in clause-boundary garden-path sentences, but ORCs prevent it. I argued that this increased revision difficulty incurred by ORCs might result from the ORC disadvantage independent of similarity-based encoding interference and/or incremental shifts in the grammatical functions of the referentially related DPs. Alternatively, the results may indicate that the parser gives weight to grammatical case during the revision of clause-boundary garden-path sentences.

## Footnotes

1 In this paper, I use labelled square brackets to describe hierarchical syntactic structures assigned during the online language comprehension process based on the *X-bar theory* (e.g., Chomsky, 1981, 1986). These syntactic structures are abstract mathematical structures that model the distribution of form and meaning by representing syntactic objects projected by each lexical item, as in Fig. 1 (for an introduction to the grammar adopted in this paper, see Chomsky, 1995; Haegeman, 1994; Hornstein et al., 2005; Lasnik & Uriagereka, 1988; van Riemsdijk & Williams, 1986)

2 The matrix subject analysis (2b) corresponds to the string of (1) because the matrix subject position must have an overt element (*[_CP_ [_PP_ After Mary dressed John] [_TP_ ε/pro/PRO bathed]], where the asterisk indicates that the structure is ill-formed), the embedded verb can be intransitive, and structurally only the locally ambiguous DP can function as the matrix subject.

3 I postulate that the embedded object analysis results from two biases. The first bias concerns the immediate incremental satisfaction of *the theta criterion* (see Chomsky, 1981; Crocker, 1996; Fujita, 2023; Gibson, 1991; Pritchett, 1988, 1992; Weinberg, 1999), and the second bias pertains to a preference to analyse an incoming element as part of the most local structure (e.g., see Frazier, 1979; Kimball, 1973; Phillips, 1996).

4 The present study assumes the following regarding the initiation and completion of the revision process: (i) Revision is initiated when a previously established dependency relation requires deformation. To wit, assuming that Dep is a set of dependency relations and i and j are distinct points within a sentence (i < j), revision is initiated at j if Dep_i_ ⊈ Dep_j_ (Sturt, 1997). (ii) Revision is complete when the parser assigns grammatical structures that correspond to the input including the disambiguating string. Dependency relations are defined as follows. Let S = w_1_, w_2_, …, w_n_ be a sentence in language L, where each w_i_ ∈ E, and E is a set of elements. w_i_ and w_j_ are in a dependency relation if either of the following conditions holds: (i) There exist elements x_i_, z_j_ in E, such that x_i_ ≠ w_i_, z_j_ ≠ w_j_, and replacing w_i_ with x_i_ requires replacing w_j_ with z_j_ for S to remain a sequence of L (i < j) (Chomsky, 1956), or (ii) w_i_ and w_j_ bear the same index (i < j ∨ j < i), e.g., there exists an index k such that w_i{k}_…w_j{k}_. Note that coindexed elements indicate covariance.

5 In this paper, I do not consider exempt reflexives (see Charnavel, 2020; Charnavel & Bryant, 2023; Charnavel & Sportiche, 2016; Drummond et al., 2011; Pollard & Sag, 1992; Reinhart & Reuland, 1993; Sloggett, 2017).

6 For example, in Fig. 1, the matrix subject DP c-commands the DP projected by the reflexive.

7 The grammar adopted in this study postulates the movement of elements (represented by traces) between certain syntactic positions (see the studies cited in footnote 1).

8 PRO is a covert DP. In (4a/b), PRO occupies the specifier position of the TP projected by “to”.

9 As suggested by a reviewer, comprehension questions that probe local ambiguity or the reflexive’s antecedent might influence the online revision process. For example, such questions may lead to distinct processing patterns compared to those observed in the current study by directing readers’ attention towards the experimental content. This aspect remains open for investigation in future research.

10 In response to concerns raised by reviewers regarding the potential influence of cut-offs on the results, I conducted additional analyses using different cut-offs (4,000 ms, 6,000 ms, 8,000 ms, and 10,000 ms) and no cut-offs. All these analyses yielded similar results.

11 In accordance with the suggestion of a reviewer, I analysed the regions following the post-disambiguating and post-reflexive regions. These analyses showed garden path effects and gender mismatch effects.

12 In accordance with the suggestion of a reviewer, as in Experiment 1, I analysed the regions following the post-disambiguating and post-reflexive regions. These analyses showed no garden path effects. At the region following the post-reflexive region, there was a significant ambiguity by gender interaction.

13 This hypothesis is predicated on the assumption that nominative Case provides reliable evidence for identifying the subject of a tensed clause, at least compared to grammatical categories. In English, it is a distributional fact that an element assigned nominative Case occupies the specifier position of a finite TP, whereas the DP category does not necessarily indicate a specific structural position.

14 The parser can locate the locally ambiguous DP either by conducting an iterative search within the local region of the syntactic tree or by directly accessing it using the grammatical category as a retrieval cue.

## Declarations

### Open Practices Statement

Analysis code, data and materials are available via the Open Science Framework at https://osf.io/78vmz/. None of the experiments were preregistered.
